# Tenosynovitis with rice body formation in a non-tuberculosis patient: A case report

**DOI:** 10.1080/03009730902931408

**Published:** 2009-09-07

**Authors:** Hiroyuki Nagasawa, Kyoji Okada, Seietsu Senma, Shuichi Chida, Yoichi Shimada

**Affiliations:** ^1^Division of Orthopedic Surgery, Department of Neuro- and Locomotor Science, Akita University School of MedicineAkitaJapan; ^2^Department of Orthopedic Surgery, Nakadori General HospitalAkitaJapan

**Keywords:** Diagnosis, MRI, rice body, tendon, tenosynovitis

## Abstract

In this report, we present a 68-year-old man with rice body formation in the flexor tendon sheath of the fingers without any inflammatory diseases such as tuberculosis or rheumatoid arthritis. The patient visited our institute in March 2004 with a one-month history of swelling and pain of the right distal forearm. Laboratory data were within normal limits, and the rheumatoid factor was negative. He had no history of tuberculosis, and the tuberculin reaction was weakly positive. Magnetic resonance (MR) images showed a mass measuring 6 cm×4 cm around the flexor tendons of the forearm. Many rice bodies had been erupted from a small hole of the fibrous wall of the mass at the time of incisional biopsy performed in June 2004. Histological diagnosis was synovitis with fibrous loose bodies. In July 2004, spontaneous ruptures of the right fourth and fifth flexor tendons occurred. Open repair was performed in August 2004. The patient regained good function of the operated fingers with no evidence of recurrence at the latest follow-up in March 2009.

## Introduction

Rice bodies which mainly consist of fibrin are occasionally observed in the joints, bursae, and tendon sheaths among patients with rheumatoid arthritis (1) or tuberculous arthritis and/or tuberculous tenosynovitis (2). However, these rice bodies in tendon sheaths are rarely seen among non-tuberculosis patients. Only two cases with rice bodies in the tendon sheaths among non-tuberculosis patients have been reported in the English literature (3,4). In this report, we describe clinical, radiological, and histopathological findings on a 68-year-old male with rice body formation in the flexor tendon sheath of the fingers without any history of inflammatory diseases.

## Case report

A 68-year-old male who had owned a bicycle shop for several decades visited our institute in March 2004 with a one-month history of swelling and pain of the right distal forearm. His dominant hand was right. On physical examination, an elastic soft mass, measuring 6 cm×4 cm, was observed on the palmar side of the right forearm. Motion of each finger was normal, but the range of motion of the wrist joint was slightly restricted. Skin color change and local heat were not observed around the mass. Laboratory data, including rheumatoid factor and C-reactive protein, were not contributory. He had no history of tuberculosis, and his tuberculin reaction on admission to the hospital was weakly positive. No hilar lymphoadenopathy was observed on a chest radiogram.

**Figure 1.**
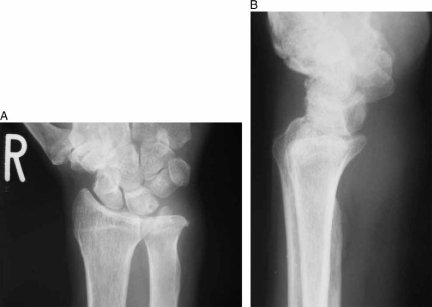
Anteroposterior (A) and lateral (B) radiogram of the right wrist shows the soft tissue mass on the palmar side without calcification.

**Figure 2.**
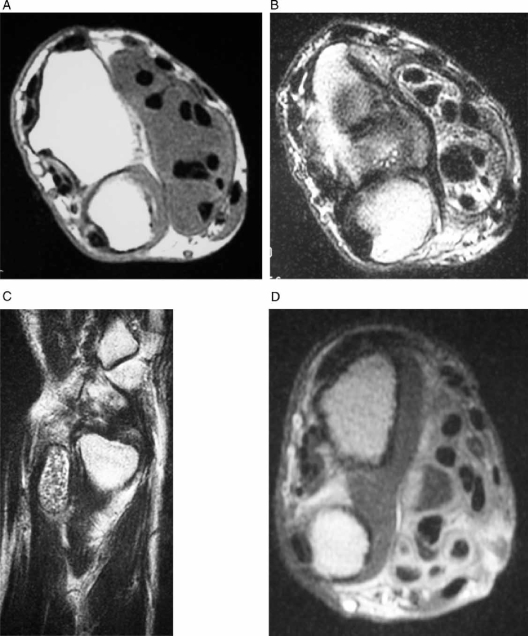
A: Axial T1-weighted MR image of the right distal forearm demonstrates a mass with low signal intensity surrounding the flexor tendons. B: Axial T2-weighted MR image showed a mass with high signal intensity mass and many rice bodies with low signal intensity. C: Sagittal T2-weighted MR image of the right distal forearm showed enlarged tendon sheaths and many small bodies with low signal intensity. D: Axial T1-weighted MR images of the right distal forearm after the administration of gadolinium. The mass which surrounded the flexor tendons was moderately enhanced.

Plain radiograms of the right wrist and hand showed degenerative changes and Heberden's nodes in the distal interphalangeal joints, but no apparent calcification was observed in the mass (Figure 1). On magnetic resonance (MR) imaging, T1-weighted images showed a low-intensity mass surrounding the flexor tendons (Figure 2A). On T2-weighed images, the mass partially showed high-signal intensity (Figure 2B). Sagittal T2-weighed images demonstrated an effusion in the sheaths of the flexor tendons and many small low-intensity bodies in the mass (Figure 2C). The mass was moderately enhanced by administration of intravenous gadolinium (Figure 2D), but the small bodies were not enhanced.

**Figure 3.**
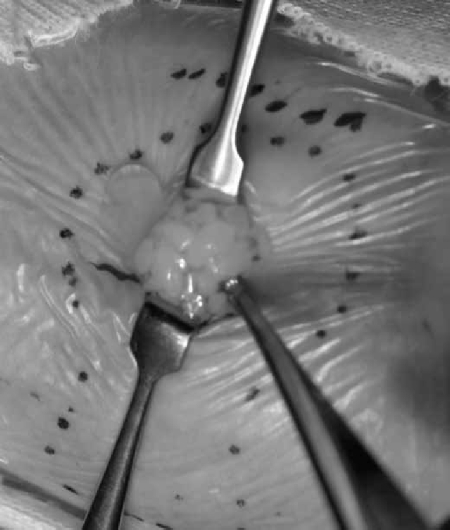
During the surgery, many rice bodies had been erupted from a small hole in the fibrous wall of the mass.

**Figure 4.**
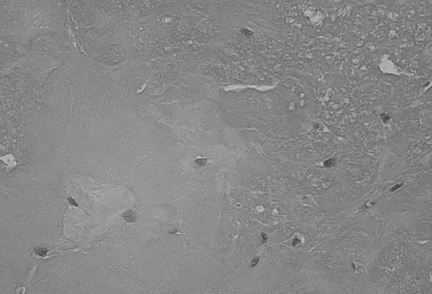
Photomicrograph of rice bodies from the distal forearm showed collagen fibers around the fibrin-like materials (×200).

**Figure 5.**
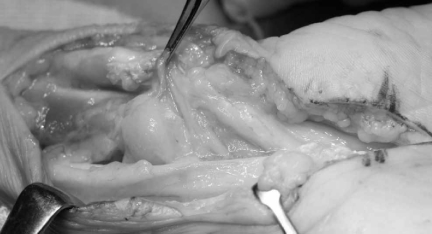
Photograph during an open repair surgery showed the granulation tissue around the flexor tendons on the volar side of the right forearm.

**Figure 6.**
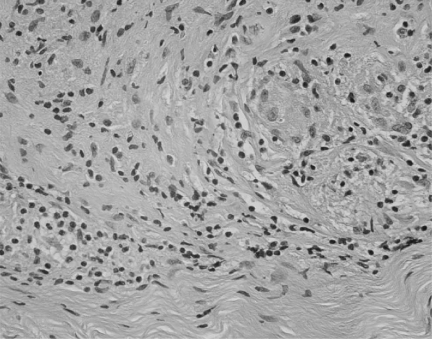
Photomicrograph of granulation tissue from the right distal forearm showed chronic synovitis with lymphoid cell infiltration. No acid-fast bacilli were found (×200).

An incisional biopsy was performed in June 2004. The mass existed adjacent to the flexor tendons and was encapsulated by a yellow fibrous wall. Many rice bodies had erupted from a small hole in the wall of the mass (Figure 3). Histological findings revealed hyperplastic tenosynovitis with fibrinous loose bodies (Figure 4). During the surgery, we confirmed that the flexor tendons were intact. In July 2004, he complained of disability to flex in his ring and little fingers. After physical examinations, we made a diagnosis of spontaneous rupture of the flexor tendons. An open repair was performed in August 2004. During the surgery, we observed granulation tissues proliferating around the flexor tendons (Figure 5) and a rupture of the flexor tendons of the ring and little fingers. The granulation tissues were removed, and the superficial flexor tendon was transferred to the ring finger. Tendon graft of the palmaris longus to the little finger was also performed. Histologically, the granulation tissues showed chronic synovitis (Figure 6), and acid-fast bacilli were negative. There was no apparent granuloma suggesting of sarcoidosis. The patient regained good function of the operated ring and little fingers with no evidence of recurrence at the latest follow-up in March 2009.

## Discussion

Rice bodies in the tendon sheaths are occasionally observed as a result of tuberculous tenosynovitis. The incidence of rice body formation is less than 50% of cases of tuberculous tenosynovitis (2). In rheumatoid arthritis, rice bodies are often observed in the subacrominal bursa but rarely observed in the tendon sheaths. Only a few cases have been reported with rice body formation in the tendon sheaths without tuberculous tenosynovitis. Muirhead et al. reported a case of a 9-year-old boy with rice bodies in the tendon sheath of the right tibialis posterior tendon subsequent to a thorn injury (3). Sugano et al. reported an 81-year-old man with rice bodies in the common flexor synovial sheath of the left wrist (4). In both cases, the rheumatoid factor was negative, and both had no history of tuberculosis. Clinical features of the present case are similar to those reported by Sugano et al.

Several authors have speculated on the nature of rice bodies. Berg et al. suggested in the electron microscopy study of rice bodies obtained from the joints of rheumatoid arthritis patients that non-vascularized rice bodies might have formed *de novo* as a part of the inflammatory reaction in the synovial fluid (1). Cheung et al. reported that the rice bodies arose from infarcted synovial cells and these cells were shed into articular or bursal fluid (5). The present patient, however, had no inflammatory disease besides localized synovitis. The mechanism of rice body formation is still obscure.

The MR appearance of rice bodies in the bursae, joints, or tendon sheaths has been described as an iso- or hypointensity with effusion on T1- and T2-weighted images (4,6,7). The rice bodies in the present case, showed similar signal intensity. However, in contrast to the case reported by Sugano et al., the mass in the current case surrounded the flexor tendons. Differential diagnosis of the mass lesions surrounding the flexor tendons includes tuberculous tenosynovitis and sarcoidosis. MRI magnetic resonance image (MRI) features of tuberculous tenosynovitis have been described as an intermediate-intensity mass on T1-weighted images and a high-intensity mass on T2-weighted images (8). In sarcoidosis, only a few reports have described the MR appearances. Katzman et al. reported a case of sarcoidosis as a high-intensity mass surrounding the flexor tendons of the wrist on T2-weighted images (9). We excluded tuberculosis and sarcoidosis from the histopathological findings including acid-fast bacilli stain.

In rheumatoid tenosynovitis, spontaneous tendon ruptures often occur in the wrist and hand due to bone and joint deformities (10). However, spontaneous rupture of the flexor tendon associated with synovitis with rice body formation has not been reported previously in the English literature. The relationship between the tendon rupture, degenerative joint diseases, and rice body formation should be further investigated.

In conclusion, we have presented the case of a 68-year-old man with rice body formation in the flexor tendon sheaths of the fingers who did not have any inflammatory diseases such as tuberculosis or rheumatoid arthritis. We should keep in mind that a spontaneous tendon rupture can occur associated with synovitis with rice body formation.

## References

[1] Berg E, Wainwright R, Barton B, Puchtler H, McDonald T (1977). On the nature of rheumatoid rice bodies: an immunological, histochemical, and electron microscope study. Arthritis Rheum..

[2] Pimm LH, Waugh W (1957). Tuberculous tenosynovitis. J Bone Joint Surg..

[3] Muirhead DE, Johnson EH, Luis C (1998). A light and ultrastructural study of rice bodies recovered from a case of date thorn-induced extra-articular synovitis. Ultrastruct Pathol..

[4] Sugano H, Nagao T, Tajima Y, Ishida Y, Nagao K, Ohno T (2000). Variation among giant rice bodies: report of four cases and their clinicopathological features. Skeletal Radiol..

[5] Cheung HS, Ryan LM, Kozin F, McCarthy DJ (1980). Synovial origins of rice bodies in joint fluid. Arthritis Rheum..

[6] Chen A, Wong LY, Sheu CY, Chen BF (2002). Distinguishing multiple rice body formation in chronic subacrominal-subdeltoid bursitis from synovial chondromatosis. Skeletal Radiol..

[7] Mutlu H, Silit E, Pekkafali Z, Karaman B, Omeroglu A, Basekim CC (2004). Multiple rice body formation in the subacrominal-subdeltoid bursa and knee joint. Skeletal Radiol..

[8] Sueyoshi E, Uetani M, Hayashi K, Kohzaki S (1996). Tuberculous tenosynovitis of the wrist: MRI findings in three patients. Skeletal Radiol..

[9] Katzman BM, Caligiuri DA, Klein DM, Perrier G, Dauterman PA (1997). Sarcoid flexor tenosynovitis of the wrist: A case report. J Hand Surg..

[10] Ferlic DC (1996). Rheumatoid flexor tenosynovitis and rupture. Hand Clin..

